# Programmed Self-Assembly of a Biochemical and Magnetic Scaffold to Trigger and Manipulate Microtubule Structures

**DOI:** 10.1038/s41598-017-10297-y

**Published:** 2017-09-12

**Authors:** Rémi Ducasse, Wei-An Wang, Marina Garcia-Jove Navarro, Nicolas Debons, Alexandra Colin, Jérémie Gautier, Jean-Michel Guigner, François Guyot, Zoher Gueroui

**Affiliations:** 1 École Normale Supérieure, PSL Research University, CNRS, UPMC, Department of Chemistry, 24 rue Lhomond, 75005 Paris, France; 2 IMPMC. Sorbonne Université. CNRS. UPMC. MNHN. IRD. 4, place Jussieu, 75005 Paris, France

## Abstract

Artificial bio-based scaffolds offer broad applications in bioinspired chemistry, nanomedicine, and material science. One current challenge is to understand how the programmed self-assembly of biomolecules at the nanometre level can dictate the emergence of new functional properties at the mesoscopic scale. Here we report a general approach to design genetically encoded protein-based scaffolds with modular biochemical and magnetic functions. By combining chemically induced dimerization strategies and biomineralisation, we engineered ferritin nanocages to nucleate and manipulate microtubule structures upon magnetic actuation. Triggering the self-assembly of engineered ferritins into micrometric scaffolds mimics the function of centrosomes, the microtubule organizing centres of cells, and provides unique magnetic and self-organizing properties. We anticipate that our approach could be transposed to control various biological processes and extend to broader applications in biotechnology or material chemistry.

## Introduction

In living systems, proteins self-organize into macromolecular assemblies at various length scales to ensure the coordination of numerous biological functions in space and time^1, 2^. For instance, at the nanometer scale, protein scaffolds are central to trigger by proximity the activation of proteins involved in signal transduction^3, 4^. At the micrometric scales, multimeric interactions or repetitive interacting domains drive the organization of numerous functional structures, such as cytoskeleton fibres or organelles, with specific functional properties usually not found at the single molecule level^2, 5, 6^. Numerous studies are now engaged to establish a clear link between biological multiscale assemblies and emergent functional properties. From this perspective the development of bio-based nanomaterials, produced from the programmed assembly of biomolecules as DNA, RNA, and proteins, offers novel tools to analyse and control the spatiotemporal properties of molecular and cellular processes, but also to engineer novel synthetic functionalities^7, 8^. For instance, DNA-based scaffolds, which provide very precise biomolecule spatial positioning, have been used to elucidate biophysical mechanisms underlying cytoskeleton motor activity^9–11^ or as fluorescent biosensors to probe the internal environment of living cells^12, 13^. Complementary, pioneer studies have demonstrated how synthetic protein scaffolds can modulate the cooperativity of ensembles of molecular motors^14^, and artificially control metabolic flux^15^ or signalling pathways^16–18^.

In this study we present a synthetic protein scaffold that combines specific features found in natural systems, such as multimeric interactions and multiscale assemblies, with novel properties provided by an artificial approach, such as stimulus-triggered assembly^19^ and magnetic control^20–27^. In this regard, our synthetic protein scaffold, bioengineered from ferritin nanocages, recapitulates several remarkable characteristics: (i) upon chemical stimulation it self-organizes into micrometric structures *in vitro*, (ii) it can be functionalized with regulatory proteins to trigger specific biochemical responses, and (iii) it displays magnetic properties that could be useful for detection or for magnetic actuation. To illustrate the versatility of our synthetic scaffold, we devised an *in vitro* assay to study specific features of cytoskeleton spatial organizations, by focusing on the nucleation and the magnetic manipulation of microtubule structures. In particular, the generation of 3D micrometric scaffolds from single functionalized and biomineralised ferritins enables us to mimic Microtubule Organizing Centres, such as the centrosome, and to examine an emergent function of these resulting artificial organelles: the centring property, which is essential to define the polarity of cells^28, 29^.

**Figure 1:**
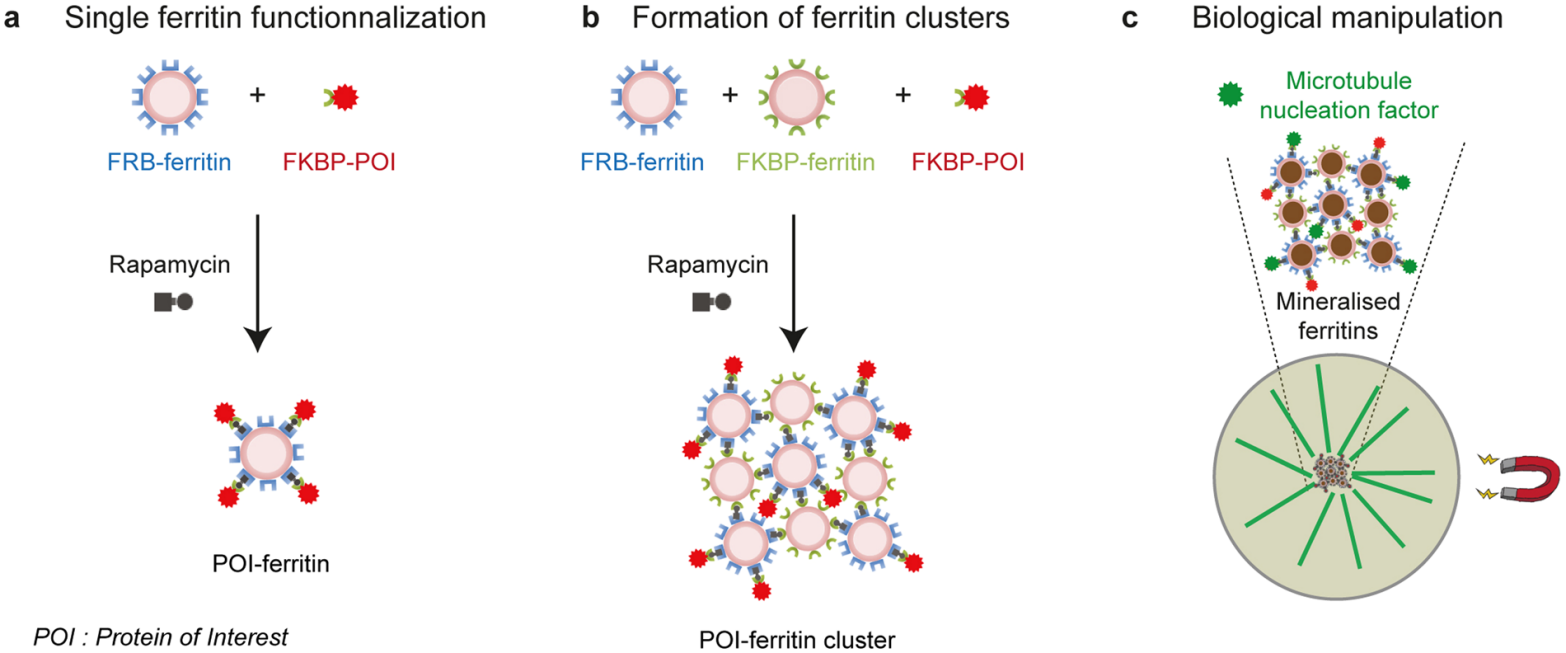
Genetically encoded protein scaffolds with modular biochemical and magnetic functions. (**a**,**b**) Schematic of the modular approach to functionalize and organize ferritin nanocages. (**a**) Chemically inducible dimerization strategy based on the heterodimerization of FKBP and FRB is used to target a protein of interest (POI) at the surface of the ferritin nanocages by addition of Rapamycin. (**b**) The formation of ferritin clusters is triggered by multimeric interactions between FKBP- and FRB-ferritins in presence of Rapamycin. (**c**) Illustration of the functionalization and magnetic manipulation of biomineralised ferritin clusters to control a microtubule assembly in space and time.

The iron storage ferritin is a protein that assembles into a nanocage composed of 24-subunits. The capacity of ferritin to catalyse the precipitation of inorganic condensed phases within its internal cavity allows living organisms to control the availability of iron^30^. Several studies have reported modifications of the ferritin cage surface by non-covalent interactions in response to electrostatic interactions^31, 32^ or metal coordination^33^. The oligomeric state of the ferritins has been further exploited to generate controlled multi-scale assemblies^31, 32, 34–36^. On the other hand, the catalytic activity of the ferritin has been used for developing novel contrast agents in living organisms (magnetic resonance imaging, electron microscopy), nanoheaters for hyperthermia, nanoprobes for biosensing and cell markers, and magnetic actuators for gene expression control (magnetogenetics)^37–46^. In this study, we exploit both the multivalent and catalytic properties of ferritin. Our first goal was to engineer ferritin nanocages as building blocks for the production of inducible micrometric protein scaffolds sharing a specific biochemical activity and magnetic properties. To do so, we have designed a strategy for the controlled functionalization of the nanocage surface, by multivalent protein-protein interactions. We have genetically modified the ferritin monomer to use chemically inducible dimerization strategies based on the heterodimerization of FKBP and FRB (Fig. 1)^47–49^. Then, the catalytic activity of ferritin, by synthesising monodispersed ferric condensed nanoparticles within its cavity, provides specific magnetic properties to the scaffold^43, 50–52^. This modular strategy permits the targeting of proteins of interest at the nanocage surface (Fig. 1a), but also the formation of 3D clusters of ferritins by triggering multimeric interactions between FKBP- and FRB-ferritin cages and magnetically manipulating them, upon biomineralisation of the participating ferritins (Fig. 1b,c).

We next examined how ferritin nanocages can serve as biochemical scaffolds to control specific biological processes using *Xenopus* egg extracts, a powerful cell-free system well suited to study the morphogenetic properties of cytoskeleton networks^53–56^. We showed that the nucleation of microtubule fibres and formation into asters can be triggered by individual ferritin nanocages when TPX2, a nucleating promoting factor, is recruited to their surface. Moreover, when organized into micrometric clusters, the TPX2-ferritin scaffolds are integrated into the microtubule asters and localized at their pole centre, which confers the ability to manipulate the aster position with magnetic forces. Our observations also suggest the emergence of different biophysical and self-organizing properties of microtubule asters, depending on the scale of ferritin organization, from single cages (nanometre) to clusters (micrometre). Finally, these results illustrate how TPX2-ferritin scaffolds share some similarities with Microtubule Organizing Centres, found in eukaryotic cells, and suggest their utilization as artificial magnetic centrosomes to manipulate microtubule-based structures.

## Results

### Modular Approach to Functionalize and Organize Ferritin Nanocages

In order to functionalize the ferritin, we used chemically inducible dimerization to target a protein of interest at the surface of the ferritin nanocages (Fig. 1a). For this purpose, we selected the proteins FRB (the rapamycin-binding domain of mTOR) and FKBP (the FK506 binding protein) that are able to heterodimerize on fast time-scales upon interaction with the molecule Rapamycin^57^. We genetically fused FRB and FKBP, respectively, to the N terminus of ferritin monomer from the extremophile archaea *Pyrococcus furiosus*. This approach offers several advantages as a result of the multivalency of the ferritin. For instance, each ferritin nanocage fused to FRB can interact with up to 24 FKBP fused to a protein of interest (and symmetrically FKBP-ferritins with up to 24 FRB-Protein of interest, Fig. 1a). This anticipates a possible control of the number of proteins being recruited per nanocage or the co-recruitment of different protein families. Furthermore, this approach can also be extended to organize micrometric clusters of protein nanocages by inducing local interactions between FRB- and FKBP-ferritin cages upon Rapamycin addition (Fig. 1b).

**Figure 2:**
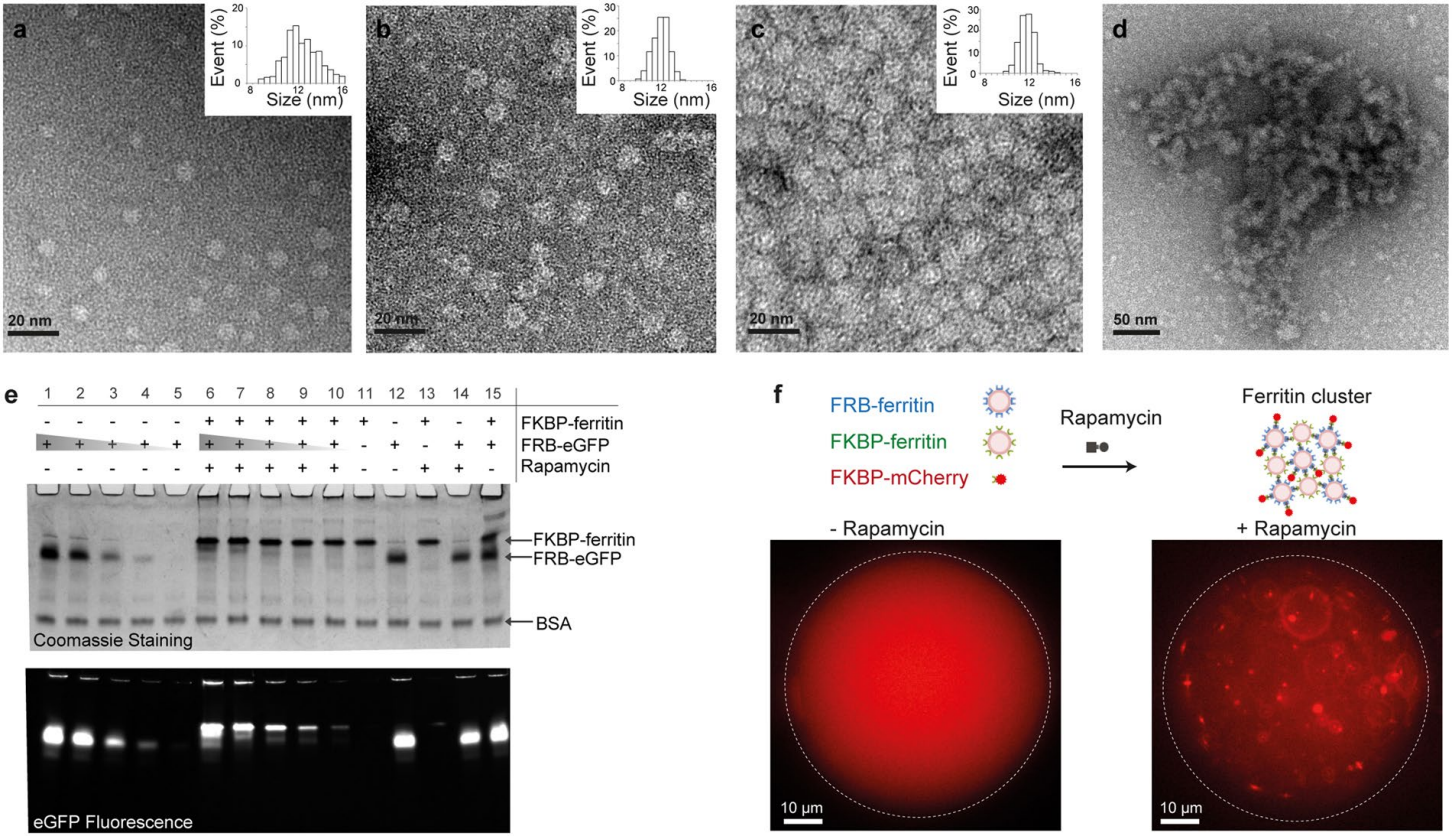
Characterisation of ferritin assemblies and biofunctionalisation. (**a**–**d**) Transmission electron microscopy of negatively stained (**a**) FRB-ferritins, (**b**) FKBP-ferritins, (**c**) native ferritins, and (**d**) ferritin clusters (inserts: size distribution histograms). (**e**) Acrylamide gel electrophoresis of FKBP-ferritins, FRB-eGFP proteins, and eGFP-ferritin complexes. From lane 11 to 15, 10 µM of FRB-eGFP and 10 µM of FKBP-ferritin migrate freely, in absence (lanes 11, 12 and 15) or presence (lanes 13 and 14) of 20 µM of Rapamycin. On lanes 1 to 5, the monomeric profile of 15–10–5–2.5–1 µM of FRB-eGFP is assessed in presence of Rapamycin. On lanes 6 to 10, according to the initial stoichiometry, the migration FRB-eGFP is retarded in presence of Rapamycin and 10 µM of FKBP-ferritin. In each well, BSA was added as loading control. (**f**) Schematic of ferritin cluster formation (top). Fluorescence images of a water droplet containing FRB-ferritins, FKBP-ferritins, and FKBP-mCherry (left). The ferritin clusters are formed in presence of Rapamycin (right).

To validate our strategy, we produced and purified recombinant FRB- and FKBP- ferritins and characterized their assembly (see Methods). Transmission electron microscopy (TEM) was used to characterize single ferritin nanocages at the nanometer scale. TEM images of negatively stained FRB-ferritins and FKBP-ferritins show a majority of monodispersed spherical objects characterized by an average diameter of 11.3 ± 1.5 nm and 12.3 ± 0.7 nm respectively (Fig. 2a,b). These data are in agreement with images acquired on native ferritins (11.5 ± 0.7 nm, Fig. 2c), suggesting that FRB- and FKBP-ferritin monomers are able to self-assemble into nanocages in solution. We performed additional physicochemical characterization of the ferritin nanocages using Dynamic Light Scattering (DLS) and zeta potential measurements (Figure S1). We found a mean hydrodynamic diameter of 13.7 nm and a zeta potential of −11.4 mV for the native ferritins; 21.9 nm and −11.1 mV for FRB-ferritins; and 18.5 nm and −5.7 mV for FKBP-ferritins. TEM images of negatively stained ferritin clusters, formed by mixing FRB-ferritin, FKBP-ferritin, and Rapamycin, revealed more complex morphologies. The clusters are organized into dense protein meshwork, as shown in Fig. 2d, and displayed a broad range of characteristic sizes (100 nm to the micrometre range, Figure S2a), corroborating with a mean hydrodynamic diameter of 215 nm associated with a large dispersion measured by DLS (Figure S1).

To confirm that chemically induced dimerization could be used to functionalize ferritin nanocages with a protein of interest, we examined the formation of complexes of FKBP-ferritin and FRB-eGFP by performing a gel mobility retardation assay (Fig. 2e). The electrophoretic migration of complexes formed by different ratios of FKBP-ferritin and FRB-eGFP proteins was analysed and compared to single protein mobility. FKBP-ferritin concentration was fixed at 10 µM, and the formation of eGFP-ferritin cages was followed in absence or presence of Rapamycin, at increasing concentrations of FRB-eGFP, to reach ratios of 1:0.1, 1:0.25, 1:0.5, 1:1, and 1:1.5 between the FKBP-ferritin monomer and FRB-eGFP (Fig. 2e). In absence of Rapamycin, no change in the migration patterns of FKBP-ferritin and FRB-eGFP was observed compared to the mobility of the free protein (Fig. 2e, lane 15 vs. control lanes 11, 12, 13, and 14). On the contrary, in presence of an excess of Rapamycin (20 µM), we noted the disappearance of the free FRB-eGFP band, both in fluorescence and Coomassie-blue observations, that was correlated with the appearance of an eGFP fluorescent signal at the level of the ferritin migration band (Fig. 2e, lane 7 vs. lane 15). Moreover, changing the FRB-eGFP concentration allowed us to tune the number of recruited proteins of interest per nanocage (Fig. 2e, lanes 6 to 10). This demonstrates the specificity and controlled binding in the recruitment of FRB-eGFP to FKBP-ferritin nanocages upon Rapamycin interaction.

Fluorescence microscopy was used to describe the behaviour of fluorescently-labelled ferritin nanocages in solution. Single FRB-ferritins were labelled by dimerizing FKBP-mCherry to the nanocage in presence of Rapamycin (Methods, Fig. 1a). The same strategy was used to label ferritin clusters: FRB-ferritin, FKBP-ferritin, and FKBP-mCherry were mixed together with Rapamycin (Methods, Fig. 2f, right). In order to facilitate the manipulation and observation, we encapsulated the ferritin constructs within water droplets dispersed in mineral oil (Fig. 2f, right). Upon clustering in presence of Rapamycin, we observed fluorescent bright dots that freely diffuse in solution (Fig. 2f, right). A very low fluorescent background noise was detected within the water droplet, suggesting that the majority of ferritin nanocages formed micrometric clusters. To the contrary, in absence of Rapamycin the fluorescent signal was homogeneous (Fig. 2f, left), thus confirming that the clustering is mediated by Rapamycin addition.

Our experiments revealed that engineered FRB- and FKBP-ferritins form monodispersed nanocages, with the capacity to be functionalized with proteins of interest at their surface, and to generate micrometric clusters in a chemically inducible manner.

### Spatiotemporal Magnetic Manipulation of Biomineralised Ferritins

**Figure 3:**
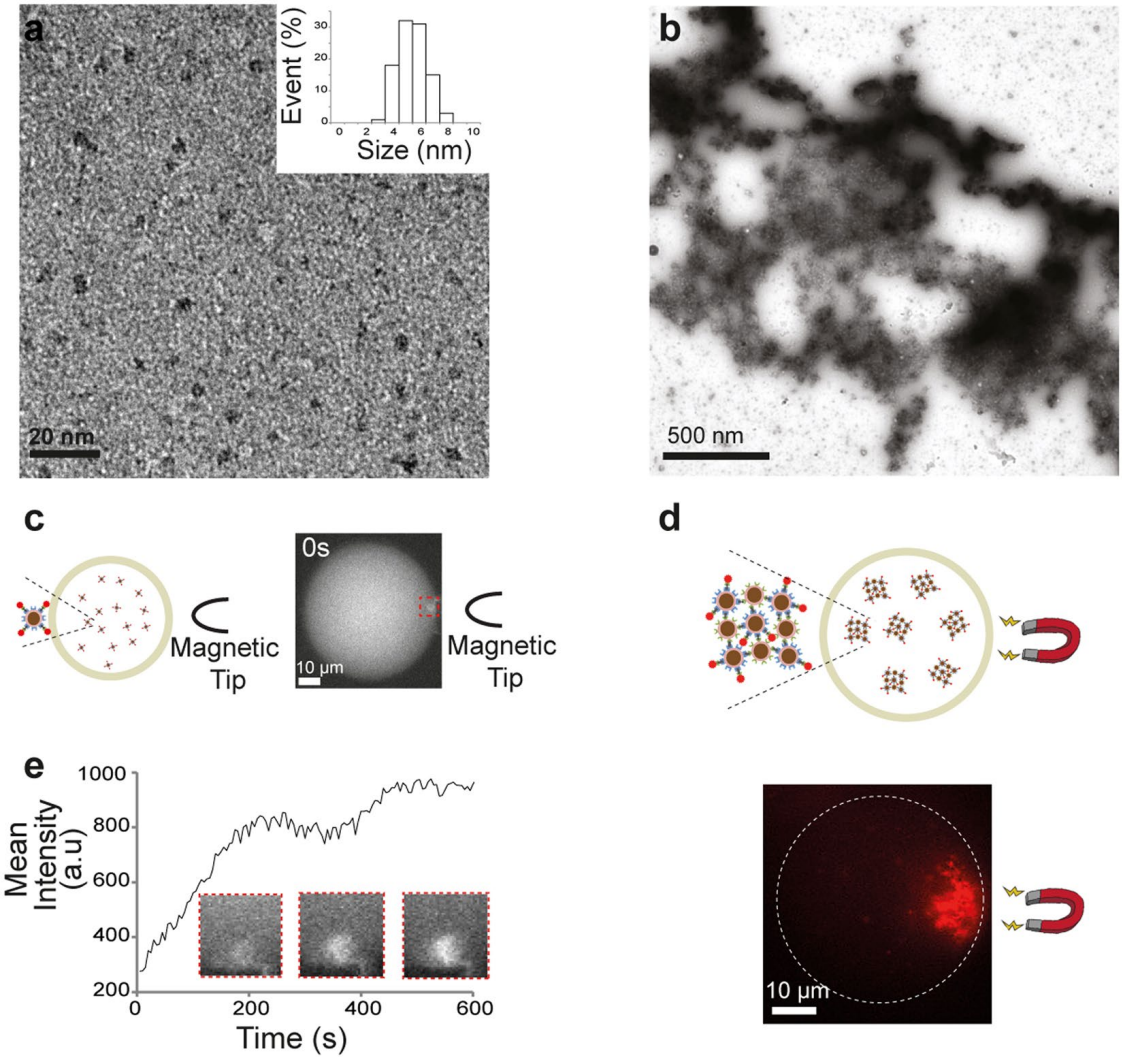
Characterisation and magnetic manipulation of biomineralised ferritins. (**a**,**b**) TEM images of mineralized FRB-ferritins and ferritin clusters (negative staining). Monodisperse iron oxide nanoparticles are shown in (**a**). The addition of Rapamycin and FKBP-ferritins into the FRB-ferritin solution causes the assembly of proteins nanocages to form micrometer-sized structures (**b**). (**c**,**d**) Fluorescence images of mineralized FRB-ferritins and ferritin clusters. The nanocages were labelled with FKBP-mCherry for fluorescence visualization within droplets of water dispersed within mineral oil (see schematic). Magnetic forces were induced by (**c**) a magnetic tip for focusing the gradient of forces, or (**d**) a permanent magnet for a long-range attraction of the clusters. (**e**) Mean fluorescence intensity as a function of time at the vicinity of the magnetic tip showing the local recruitment of single ferritins.

In order to provide magnetic properties to our engineered ferritins, the biomineralisation of iron oxide nanoparticles into ferritin cavities was performed by loading iron into FRB- and FKBP-ferritins within a controlled chemical environment (see Methods). TEM images of the mineralized FRB- and FKBP-ferritins revealed the presence of monodispersed iron oxide nanoparticles with an average size of 5 ± 1 nm (Fig. 3a). We also performed TEM on clusters assembled from interacting FRB- and FKBP-ferritins in presence of Rapamycin (Figs 3b and S2b), which permitted the confirmation of the presence of iron oxides within the clusters using scanning transmission electron microscopy (STEM) coupled energy dispersive X-ray analysis (EDX, Figure S2c,d).

To experimentally test the possibility to spatiotemporally manipulate biomineralised ferritins, magnetophoretic experiments were conducted on both single and clustered ferritin nanocages (Fig. 3c,d). The FRB-nanocages were fluorescently labelled with FKBP-mCherry by adding Rapamycin and observed within droplets of water dispersed in mineral oil to facilitate their magnetic manipulation (Fig. 3c). To estimate the effect of magnetic forces on ferritins, a gradient of magnetic field was produced by a permanent magnet (NdFeB magnet, 0.8 T, 10^2^ T.m^−1^) positioned at a distance ranging from 50 to 500 μm from the droplets; or by using a micromanipulated magnetic tip to locally enhance the gradient of magnetic field up to 10^4^ T.m^−1^. Upon the application of a magnetic field gradient of 10^2^ T.m^−1^, we observed a homogenous concentration of mCherry-labelled and mineralized ferritins within the droplets, indicating that the magnetic forces had no effect on single ferritin cages. The magnetic forces acting of single ferritins were plausibly not strong enough to overpass thermal forces, which is consistent with the weak value of the magnetic moment of the condensed phase forming the ferritin core. However, increasing the gradient up to 10^4^ T.m^−1^ with a magnetic tip allowed the local recruitment of individual mCherry-labelled ferritins induced by magnetic forces that were estimated to be in the femto-Newton range (Method, Fig. 3c). The accumulation of mineralized ferritins occurs within 3 minutes and leads to an increase of ferritin concentration at the droplet boundary (Fig. 3c). On the other hand, the magnetic manipulation of clusters of mineralized ferritins was possible at a magnetic field gradient of 10^2^ T.m^−1^ (Fig. 3d). Indeed upon magnetic field actuation, we detected a strong accumulation of the bright fluorescent clusters along the magnetic field gradient. It shows that the overall resulting magnetic forces experienced by each ferritin forming the clusters can overcome the thermal forces and the viscous drag force opposing the cluster mobility.

Overall, our data demonstrate that the magnetic manipulation of biomineralised ferritins is possible. Scaling up the hierarchical organization of the mineralized ferritins permits the demonstration of different behaviours under magnetic field actuation: while the local recruitment of single ferritins implies working in the range of few micrometres from the magnet, the complete asymmetry of clusters of ferritins within the droplets is obtained over distances of the range of 100 µm.

### Ferritin Clusters as Biochemical and Magnetic Scaffolds to Nucleate and Manipulate Microtubules

**Figure 4:**
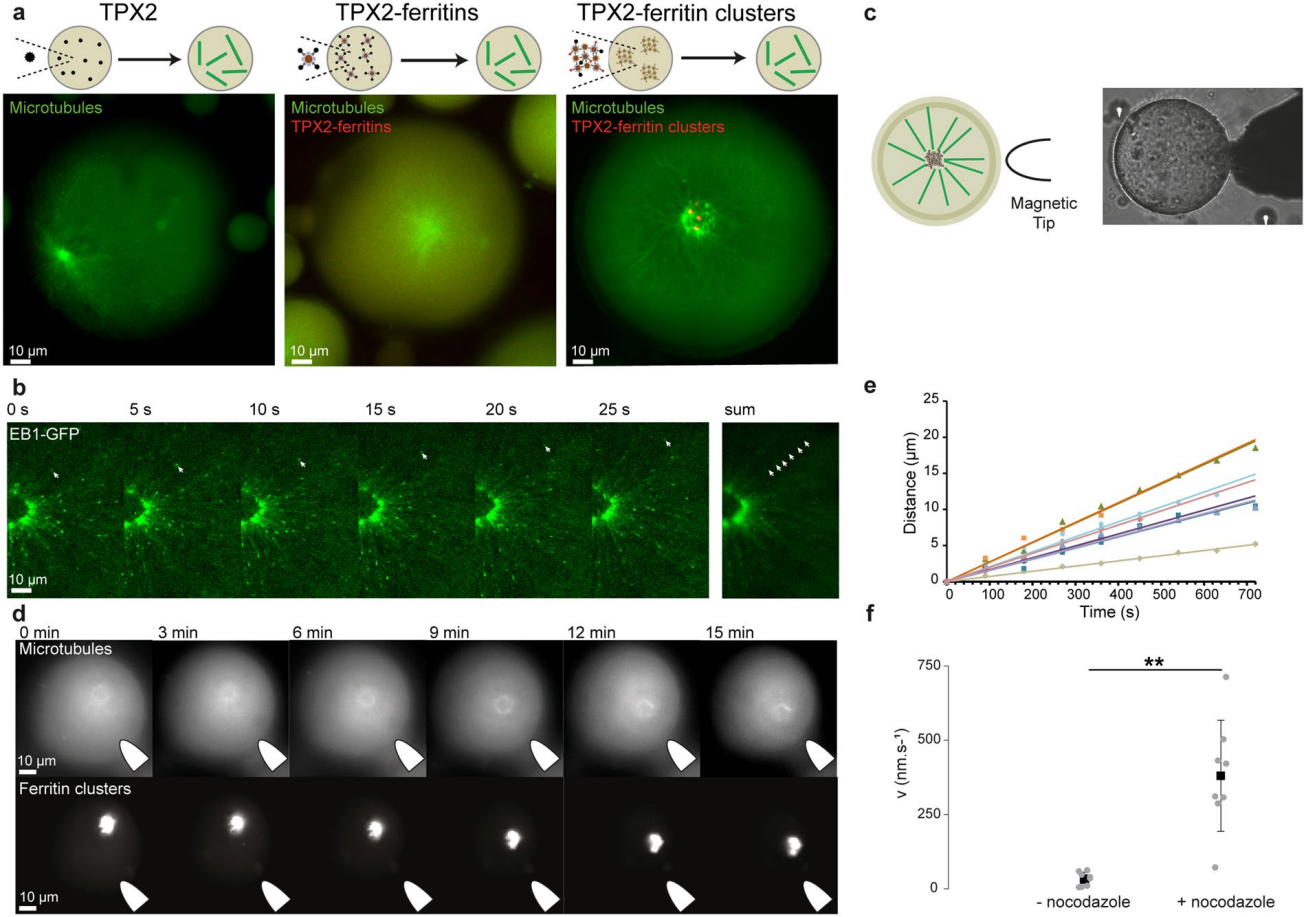
Nucleation and spatiotemporal manipulation of microtubules assemblies upon magnetic actuation. (**a**) Confocal observation of microtubule polymerization induced by FKBP-TPX2 in a droplet of *Xenopus* egg extract. Microtubules and ferritins are labelled with fluorescein-labelled tubulin and mCherry, respectively. From the left to right, microtubule structures triggered with FKBP-TPX2, mCherry/TPX2-ferritins, and ferritin-TPX2 clusters. (**b**) Time-lapse of microtubule growth polymerizing from an aster triggered by ferritin-clusters. EB1-GFP is used as fluorescent reporter of the plus-end microtubule growing extremity. **c**, Schematic of the magnetic control experiment (left). Bright-field observation of the magnetic tip positioned next to an egg extract droplet (right). (**d**) Representative time-points of the dynamic of an aster centre upon magnetic field actuation. (**e**) Example of the spatiotemporal dynamics of aster centres toward the gradient of magnetic field. Plot of the length of the trajectory of the aster as function of time when submitted to magnetic forces. (**f**) Mean velocity of the asters attracted toward the magnet in absence and in presence of a microtubule-depolymerizing drug (nocodazole).

We next investigated how to trigger a specific biological activity using ferritin nanocages as biochemical scaffolds. As proof-of-concept, we focused on the protein TPX2, which nucleates microtubule polymers^58^. The activity of TPX2-ferritin bioconjugates was assessed by monitoring their capacity to nucleate microtubules. We used *Xenopus* egg extracts, a cell-free system well-suited to examine cytoskeleton processes, encapsulated within droplets dispersed in mineral oil to mimic the cell boundary and facilitate observations^59^. In this assay, the incubation of TPX2 (0.5 µM) and fluorescently labelled tubulin (200 nM) within the egg extracts leads to the formation of fluorescent microtubules that assembled into characteristic asters (Fig. 4a, left).

As a first step, we tested the capacity of single functionalized ferritins to nucleate microtubules. Chimeric mCherry/TPX2/ferritin nanocages were produced by dimerizing both FKBP-TPX2 and FKBP-mCherry with FRB-ferritin in presence of Rapamycin (Methods). When added to the cell extract at a final concentration of 0.5 µM, the chimeric ferritins nucleate microtubules, demonstrating that TPX2 activity is maintained when dimerized with ferritin nanocages (Fig. 4a, centre). The homogeneous mCherry signal observed within the droplets indicates no permanent colocalization between TPX2 and microtubules, and suggests that the nucleation of the fibres is probably induced by transient interactions between TPX2 and tubulin.

Then, to assess the activity of the micrometric ferritin scaffolds, we assembled clusters of mCherry/TPX2-ferritins by dimerizing FRB-ferritin with FKBP-ferritin, FKBP-mCherry, and FKBP-TPX2 in presence of Rapamycin. When confined within the egg extracts, TPX2-ferritin clusters were also biochemically active and nucleate microtubule asters (Fig. 4a, right). Interestingly, the morphologies of the asters triggered by single ferritin nanocages and those triggered by ferritin clusters were different. When microtubules nucleated from TPX2-ferritin clusters, the centre of asters colocalised with a micrometric spherical meshwork of microtubules which entrapped the clusters of ferritins (Fig. 4a, right, and Figure S4). Clusters of TPX2-ferritins are probably transported during microtubule organization into radial arrays by minus-end-directed dynein motors, which are known to be present in extracts and can convey microtubule seeds. Furthermore we observed a correlation between the diameter of the aster pole entrapping the clusters of ferritins and the droplet diameter (Figure S3). To examine more closely microtubule organization and polarity, we performed confocal microscopy observations using the protein EB1-GFP, which interacts with microtubule plus-end extremities (Fig. 4b). At the vicinity of the centre of the asters, the growth of the microtubules was directed outward with a mean velocity of 15 ± 5 µm.min^−1^ (N = 7), indicating that the growing fibres have their plus-ends pointing towards the droplet boundary. Therefore the scaffold-based ferritin asters have the same polarity as acentrosomal or centrosomal asters found in living systems^28^.

Finally, we assessed the possibility to spatially manipulate the clusters of mineralized ferritins integrated to the microtubule asters with magnetic forces. In order to localize and direct the forces acting on the clusters, we used a magnetic tip that generates a focalized magnetic field gradient (Method, diameter 80–100 µm, Fig. 4c). Remarkably, when the micrometric ferritin scaffolds were entrapped in the centre of the aster, the whole microtubule based-structure was attracted along the gradient of magnetic field with an average velocity of 33 ± 23 nm.s^−1^ (Fig. 4e,f). The average velocity of the moving aster increased to around 380 ± 186 nm.s^−1^ if a microtubule-depolymerizing drug is added to the extract (nocodazole, Method, and Fig. 4f). Using Stokes’ law, we estimated that the forces acting on ferritin asters were in the Pico-newton range (Method). The large increase in velocity in absence of microtubules can be explained by the reduced friction forces experienced by the ferritin clusters in motion in comparison to the friction of the whole aster-like structures. Additionally, microtubules contribute as rigid and elastic elements that can counterbalance the applied magnetic forces.

Altogether these data demonstrate that engineered ferritin nanocages can be used as biochemical and magnetic scaffolds to trigger and localize cellular processes. In our case, micrometric magnetic scaffolds of ferritins coupled to TPX2 enable the nucleation and the manipulation mesoscopic microtubule assemblies within a confined environment.

### Ferritin Scaffolding mimics the Microtubule Organizing Centres of cells

**Figure 5:**
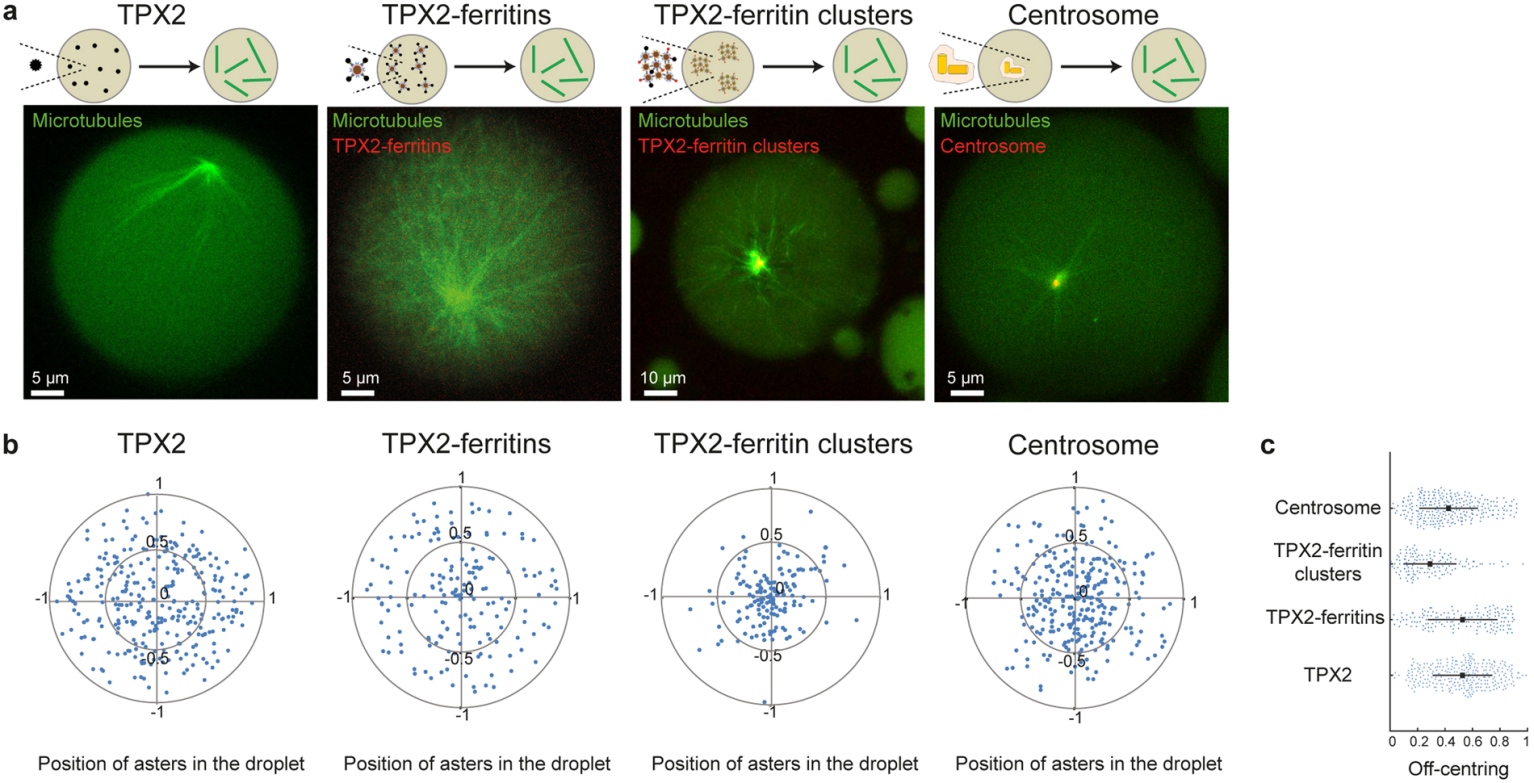
Ferritin Scaffolding mimics the positioning of Microtubule Organizing Centres of cells. (**a**) Representative observations of microtubule aster organization triggered by FKBP-TPX2, TPX2-ferritin, TXP2 ferritin-clusters, and centrosomes. Microtubules and ferritins are labelled with fluorescein-labelled tubulin and mCherry, respectively. (**b**) Geometrical positioning of asters nucleated by FKBP-TPX2, TPX2-ferritin, TXP2 ferritin-clusters, and centrosomes. 0 and ±1 indicate the centre and the droplet boundary, respectively. (**c**) Mean and standard deviation of the off-centring of the aster positions triggered by FKBP-TPX2, TPX2-ferritin, TXP2 ferritin-clusters, and centrosomes.

The functional properties of TPX2-ferritin clusters share some similarities with the Microtubule Organizing Centres (MTOCs) of eukaryotic cells, structures that nucleate microtubules and participate in intracellular spatial organization. For instance, the geometrical positioning of MTOCs is important for defining the polarity of cells and depends on the interplay between internal and external cues together with the physical properties of their constituents^60, 61^. To gain insights into the self-organizing properties of our artificial MTOCs, we quantitatively characterized the geometrical positioning of asters nucleated by single TPX2-ferritins and TPX2-ferritin clusters to compare their centring properties (Fig. 5). The asters triggered by single TPX2-ferritins have their centre distributed throughout the entire droplet space (Fig. 5a,b, TPX2-ferritins). In this case, the normalized standard deviation (SD) and average off centring (OC) of the aster positions were respectively 41% and 53%; where 0% and 100% indicate the centre and the droplet boundary, respectively (Fig. 5b). These observations were similar to those obtained for asters triggered by free TPX2 proteins (SD = 40% and OC = 54%) (Fig. 5a,b, TPX2). Remarkably, the asters triggered from micrometric scaffolds of TPX2-ferritin clusters have their centre restricted to a smaller area around to the geometrical centre of the droplet (SD = 25% and OC = 29%) (Fig. 5a,b, TPX2-ferritin clusters). This suggests that the mode of spatial organization of TPX2-ferritins, at the single nanocage level or assembled into micrometric scaffolds, directly influences the geometrical positioning of the asters during microtubule self-organization. To quantify the centring properties of asters nucleated from natural MTOCs, we next examined the positioning of asters nucleated by centrosomes purified from Jurkat cells. Interestingly, the position of the asters nucleated from a centrosome was also restricted to the vicinity of the droplet centre (SD = 32% and OC = 42%, Fig. 5a,b, Centrosome). This observation highlights that natural MTOCs have centring properties intermediate between single TPX2-ferritins and scaffold-based ferritin clusters.

It has been established that the pulling and pushing forces generated by microtubules and motor-dependent microtubules, as well as the mechanical properties of the cell boundary, participate in the overall MTOC centering^60^. We used this framework to interpret our observations and assume that the centring properties of the asters is due to pushing forces exerted by microtubules on the droplet boundary or on the extract cytoplasm. In this picture, the larger off-centre behaviour observed for the asters triggered by single TPX2-ferritins can be induced by pushing forces produced by asymmetrically oriented microtubules. It has been previously proposed that this situation arises from the pivoting of microtubule fibres around the aster pole and favour asymmetrically oriented microtubule asters^59^. On the other hand, the restricted off-centring of asters triggered by micrometric ferritin clusters could arise from microtubules that do not pivot around the aster pole^59, 61^. As the aster centre is composed of a matrix of ferritin clusters embedded within a microtubule meshwork, it may anchor microtubules. The resulting anchorage introduces a rotational stiffness resisting to the torque produced by microtubules, and maintains the fibre orientation that balances the forces positioning the aster centre.

Altogether our data demonstrates the emergence of different biophysical properties in the centring of microtubule asters, depending on the scale of ferritin organization, from single cages (nanometre) to clusters (micrometre). Furthermore, the incorporation of TPX2-ferritin scaffolds at the aster pole modifies the overall centring properties, which highlights the importance of the local organization of the aster pole for its biological function. These results illustrate how TPX2-ferritin scaffolds can mimic Microtubule Organizing Centres of eukaryotic cells with specific self-organizing properties.

## Conclusion

Overall, we have demonstrated that engineered ferritin nanocages can be designed as biochemical and magnetic scaffolds to nucleate and manipulate mesoscopic bioassemblies *in vitro*.

As magnetic interactions can be contactless, remote controlled, and can deeply penetrate into thick materials; there is a strong interest in using tailored and functionalized magnetic nanoparticles to actuate biological processes upon magnetic actuation^62–64^. Integrating such magnetic properties to bio-based scaffolds could open numerous perspectives in nanomedicine, biotechnology, or material chemistry. Our study demonstrates that protein-based magnetic scaffolds can be used to spatiotemporally manipulate biomolecules with magnetic actuation. Our scaffold combines the advantages of being genetically encoded with a synthetic approach, ensuring their modular biofunctionalisation, biocompatibility, inducible self-assembly properties, which overall provides novel opportunities alternative to the use of synthetic magnetic nanoparticles.

Our study, aiming to assemble an artificial and magnetic centrosome, raises interesting questions about the impact of multiscale assemblies on functional properties. For instance, we show that the mode of spatial organization of TPX2-ferritins, at the single nanocage level or assembled into micrometric scaffolds, influences the centring properties during microtubule self-organization, which eventually can impact the polarity of the overall cytoskeletal organization. This suggests that the fundamental property of the centring of microtubule asters may also depend on the local organization of the aster pole centre. Furthermore, our modular approach is generic and shows how artificial protein-based organelles can be engineered with emergent functional properties, suggesting its extension to mimic other protein-based organelles or to artificially trigger signalling pathways.

Finally, the transposition of our strategy into living cells will be powerful to target and manipulate specific proteins and cellular organelles by combining chemically induced dimerization and magnetic manipulation^23, 63, 65^. A fully-genetically encoded strategy, avoiding *in vitro* biomineralisation and injection into cells, will require novel approaches to catalyse enhanced magnetic phases within cells. This will be necessary to overcome the limited magnetic properties of *in vivo* expressed ferritins that is a bottleneck for efficient magnetic manipulation^66, 67^. Beyond the development of new methods for spatiotemporal control, our strategy could also benefit other applications including biomedical imaging, biosensing, drug delivery, or the design of stimuli-responsive materials.

## Methods

### Reagents

Buffers powders, ATP, creatine phosphate, creatine phosphokinase, Nocodazole, H_2_O_2_, FeSO_4_, Sodium citrate, DTT and mineral oil (M5904) were purchased from Sigma-Aldrich (St Louis, MO). Poly(12-hydroxystearic acid) (PHS) and poly(ethylene oxide) (PEO-30) are commercially available (Arlacel P135) and were purchased from UNIQEMA. Tubulins, labelled with Rhodamin or with FITC, were ordered from Cytoskeleton Inc. (Denver, CO). Rapamycin was purchased from Calbiochem. Jurkat centrosomes were kindly provided by M.Thery and J.Sillibourne. Cytostatic-factor-arrested (CSF) *Xenopus laevis* extracts were prepared as previously described^68^. All reagents for buffer preparation were purchased from Sigma-Aldrich.

### Expression and purification of recombinant proteins

The plasmid for Escherichia coli expression of the wild type ferritin (*Pyrococcus furiosus*) is a gift from W.R Hagen^69^. FKBP- and FRB-ferritin, mCherry, eGFP and xlTPX2dNLS fusion proteins were cloned in a pET28 plasmid. xlTPX2dNLS is the *Xenopus laevis* TPX2 with a mutation at residue K284 that disrupt importin-α binding and efficiently induced aster formation^70^. EB1 protein (*Xenopus laevis*) fused to GFP was expressed from a pET21a plasmid. FKBP- and FRB-ferritin, mCherry, eGFP and TPX2 recombinant proteins were produced in Rosetta(DE3)pLysS cells (Merck Millipore) by addition of IPTG (Sigma) and overnight expression. Purification of recombinant proteins was realized using standard his-tagged protein purification protocols.

### Native gel retardation assay

After centrifugation (10^4^ rpm, 5 minutes, 4 °C), different concentrations of FRB-eGFP recombinant protein (15; 10; 5; 2,5; 1 µM) were incubated during 5 minutes at room temperature in presence of 10 µM of recombinant FKBP-ferritin (monomer concentration) and 20 µM of Rapamycin (Calbiochem), in PBS pH 7.4. In each condition, 100 ng of BSA (Bio-Rad) were added as loading control. The electrophoretic mobility of the complexes formed was assed using non-denaturing 7,5% acrylamide gels (Bio-Rad) in CHES buffer pH 9.3. Protein mobility was first analysed by detecting the fluorescence of the eGFP fusion protein (Gel Doc XR + System, Bio-Rad), followed by a coomassie staining for the visualization of the total protein pattern in the gel.

### Biomineralisation of ferritins

The iron oxides nanoparticles were prepared as previously described^71^. Briefly, purified FRB/FKBP-ferritins (24 µM) were mineralized at 65 °C in 5 mM HEPES solution. Solution of Ammonium Iron (II) Sulfate hexahydrate was used as iron source. All solutions were degassed with argon and the pH was maintained dynamically at 8.5 using an automatic titrator (718, Titrator, Metrohm). Fe(II) was added to attain a loading factor of 3000 Fe per protein cage. The Fe(II) and H_2_O_2_ (3:1) were added simultaneously at constant rate of 15 µL.min^−1^ using a syringe pump (Kd scientific). The reaction was considered complete 3 hours after addition of all iron and oxidant solutions. The mineralized sample was dialyzed overnight against 5 mM HEPES solutions.

### Chimeric ferritin assembly and cluster formation

Proteins were first sonicated (5 minutes) and centrifuged (10^4^ rpm, 5 minutes, 4 °C) to remove aggregates. Proteins and Rapamycin were then mixed in HEPES buffer with an incubation time of 5 minutes at room temperature. Single FRB-ferritins were labelled by dimerizing FKBP-mCherry to the nanocage in presence of Rapamycin with a ratio of 12:1 between monomers and mCherry. The ferritin clusters were formed by mixing FRB-ferritin, FKBP-ferritin, and FKBP-mCherry together with Rapamycin at a ratio of 24:5:2 between monomers and mCherry. Ferritin monomers and TPX2 were mixed at a ratio of 1:0.1 to 1:0.25.

### Transmission electron microscopy

Transmission electron microscopy (TEM) was performed at 200 kV using a Jeol 2100 equiped with a LaB6 gun and a Jeol 2100 F with a field emission gun (FEG). Both microscopes had energy dispersive x-ray (EDX) detectors for analyzing chemical compositions. Samples were prepared on carbon-coated copper grids by placing 4 µL drop solution containing the ferritin solution. On the LaB6-TEM, we studied samples deposited on carbon-coated copper grids negatively stained using an aqueous solution of uranyl acetate at 2% in mass. On the FEG-TEM, we studied samples deposited on carbon-coated copper grids without staining. Iron mineralization was studied by EDX spectra and maps acquired in scanning transmission electron microscopy (STEM).

### DLS and Zeta potential characterization

The DLS measurements were performed using a Brookhaven Instruments System (BI-200SM goniometer, BI-9000AT correlator, equipped with a 30 mW laser diode operating at a wavelength of 637 nm). The Size distribution of scatterers was determined by NNLS procedures. Zeta potential of the prepared protein nanoparticles were measured using a Zetasizer Nano ZS (Malvern Instruments Ltd., UK). Each sample was dissolved in PBS buffer in presence of 10 mM KCl at concentration of 0.2–1.0 mg/ml.

### Microtubule assembly

Microtubule structures were assembled using metaphase *Xenopus laevis* egg extracts, containing an ATP regenerating system (1 mM ATP, 10 mM creatine phosphate, 100 μg/μL creatine phosphokinase, final concentrations), incubated in the presence of 500 nM of FKBP-TPX2 for 30 min at 19 °C. Microtubules were labelled either with Rhodamin-labelled tubulin at 100 nM final (Cytoskeleton Inc.), fluorescein-labelled tubulin at 100 nM final (Cytoskeleton Inc.), or EB1-GFP at 150 nM final. Microtubules were disrupted using nocodazole at a final concentration of 10 µM.

### Extract-in-Oil Droplet Formation

Cellular extract was encapsulated in droplets via water-in-oil emulsion process. Mineral oil contains a biocompatible block copolymer in order to stabilize emulsion and facilitate observations. This method has been previously described and allows the formation of microtubule asters in droplets^59^. Preformed protein clusters mixed to the *Xenopus laevis* egg extracts containing fluorescently labelled tubulin were added to the block copolymer solution (1% (v CSF/v Oil)) at room temperature. The mixture was then gently sheared, by pipetting up and down the solution during few seconds, to generate extract-in-oil droplets. The mechanical dispersion of the biphasic solution formed micrometre-sized extract-in-oil droplets within few seconds. The emulsion was incubated for 20–30 minutes at 19 °C.

### Imaging and data analysis

Fluorescence imaging of microtubule asters was performed using an IX81 (Olympus) and ×60 (PlanApo, NA 1.42) oil objective, equipped with an EM-CCD camera (electron multiplying CCD, C9100-02, Hamamatsu, Corporation), and a LED system of illumination (Spectra X, Lumencor). Microscope settings and functions were controlled using Simple PCI software (Hamamatsu). Image analysis was performed using and Simple PCI and ImageJ. Confocal microscopy was performed with a Zeiss LSM 710 META laser scanning confocal microscope using ×63 (PlanApochromatic, NA 1.4) objective. Image analysis was performed using LSM Software Zen 2009 and ImageJ. Student’s t-tests and the interpretation of the p-values: NS means there is no significant difference between the two distributions. One star means pvalue < 0.05, two stars means pvalue < 0.01, three stars means pvalue < 0.001.

### Magnetic manipulation of structures

Two types of magnetic manipulations were done. First, a permanent NdFeB magnet (3 mm length, W-03-N Supermagnete, 0.8 T, 10^2^ T.m^−1^) was placed next to oil droplets. Second, a magnetic tip was elongated from a guitar string leading to a radius of curvature of 25 µm. The tip was then adapted on the N-S axis of a NdFeB permanent magnet (3 mm length) and placed next to oil droplets using a manual micromanipulator (Narishige). With this system the gradient of magnetic field can reach 10^4^ T.m^−1^ at the vicinity of the tip.

The magnetic force exerted on a ferritin is the spatial derivative of the magnetic energy −1/2 μB * B, µ is the magnetic susceptibility of a ferritin particle: $F=m\frac{dB}{dx}\approx 1\,fN$, with *m* the magnetic moment of the ferritin (10^−19^ A.m^2^) and $\frac{dB}{dx}$ the magnetic gradient (10^4^ T.m^−1^). This value is consistent with the one estimated from B, and the volume and the magnetic susceptibility of the ferritin particle:

*F* = *V* * χ/µ_0_ $\ast B\ast \frac{dB}{dx}$ ≈ 1 *fN*. Consequently the force acting on a cluster formed of 10^4^ ferritins is about *F*_*cluster*_ = *N* * *F* ≈ 10 *pN*.

The forces acting on ferritin-asters were calculated using Stokes’ law. We first estimated the forces applied on clusters of ferritins with our magnetic assay using *Xenopus* egg extract supplemented by nocodazole to recapitulate the viscous nature of the extracts and rule out the elastic forces opposing the magnetic forces that could provided an array of microtubules. We find forces of about 2 *pN*. This result was confirmed when computing directly the force acting on moving aster when taking into account the effective size of the radial organization.

## Electronic supplementary material

Supplementary Information
