# Supplementary material for: Programmed Self-Assembly of a Biochemical and Magnetic Scaffold to Trigger and Manipulate Microtubule Structures
Source: Sci Rep. 2017 Sep 12;7:11344. doi: 10.1038/s41598-017-10297-y (PMC5595911; doi:10.1038/s41598-017-10297-y)
Supplement: Supplementary file 1 — Supplementary Information [file 41598_2017_10297_MOESM1_ESM.pdf]

Supplementary Information

Programmed Self-Assembly of a Biochemical and Magnetic Scaffold to Trigger and Manipulate Microtubule Structures

Rémi Ducasse[1], Wei-An Wang*[1], Marina Garcia-Jove Navarro*[1], Nicolas Debons[1], Alexandra Colin[1], Jérémy Gautier[1], Jean-Michel Guigner[2], François Guyot[2], and Zoher Gueroui[1]*

[1] École Normale Supérieure, PSL Research University, CNRS, UPMC,
Department of Chemistry,
24 rue Lhomond, 75005 Paris, France

[2] IMPMC. Sorbonne Université. CNRS. UPMC. MNHN. IRD.
4, place Jussieu, 75005 Paris, France

* R.D, WA.W, and M.GJ.N contributed equally to this work.

4 Supplementary figures.

Correspondence:
Zoher Gueroui
Phone: +33 1 44 32 24 09
Email: zoher.gueroui@ens.fr

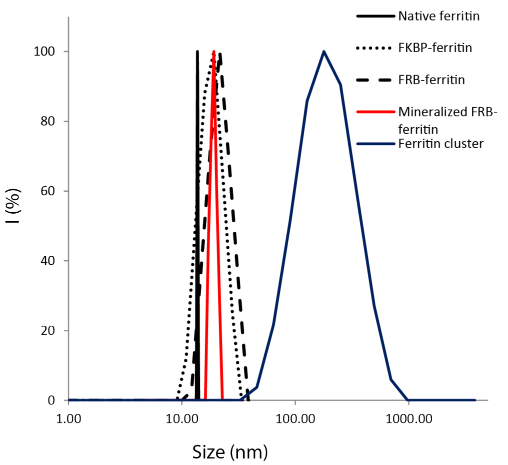

Protein	mean size (nm)[a]	zeta potential (mV)[b]
Native ferritin	13.7 ± 0.55	-11.4
FKBP-ferritin	18.5 ± 8.5	-5.7
FRB-ferritin	21.9 ± 9.8	-11.1
Mineralized FRB-ferritin	19.1 ± 2.7	-1.5
Ferritin cluster	215.1 ± 279.8	-13.7

Figure S1. Size distribution of native ferritin, FKBP-ferritin, FRB-ferritin, mineralized FRB-ferritin and ferritin cluster determined by dynamic light scattering (DLS).
Table: Diameter and zeta potential of native ferritin, FKBP-ferritin, FRB- ferritin, mineralized FRB-ferritin, and ferritin cluster. [a] Mean diameter measured by dynamic light scattering (DLS) and [b] Zeta potential.

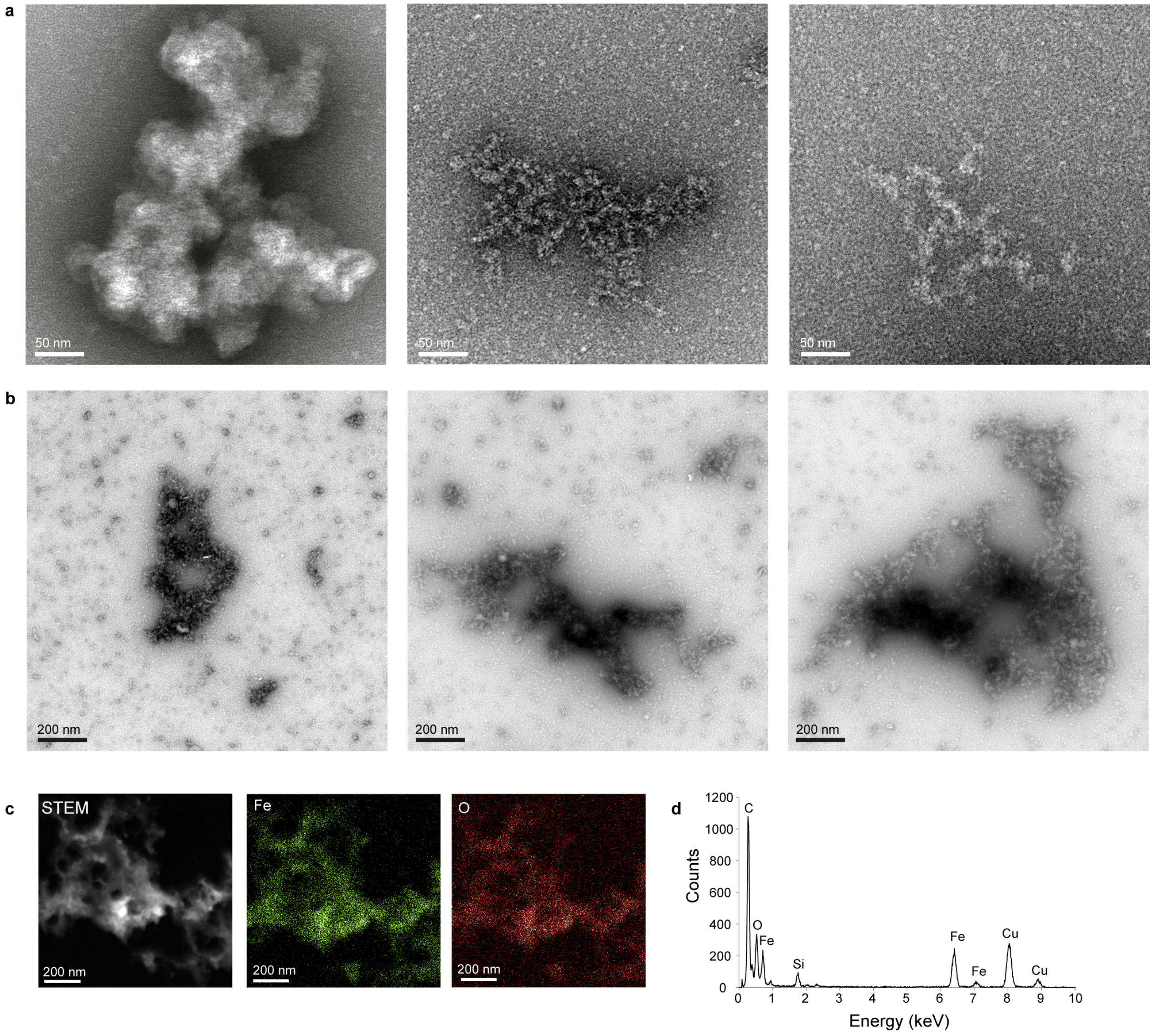

Figure S2. Characterization of ferritin assemblies and chemical analysis of biomineralised ferritin clusters: **a,b,** TEM negatives staining images of ferritin clusters and mineralized ferritin clusters. **c,** STEM-EDX mapping of the ferritin cluster showing the distribution of Fe (green) and O (red) within the ferritin cluster. **d,** EDX spectrum of the ferritin cluster.

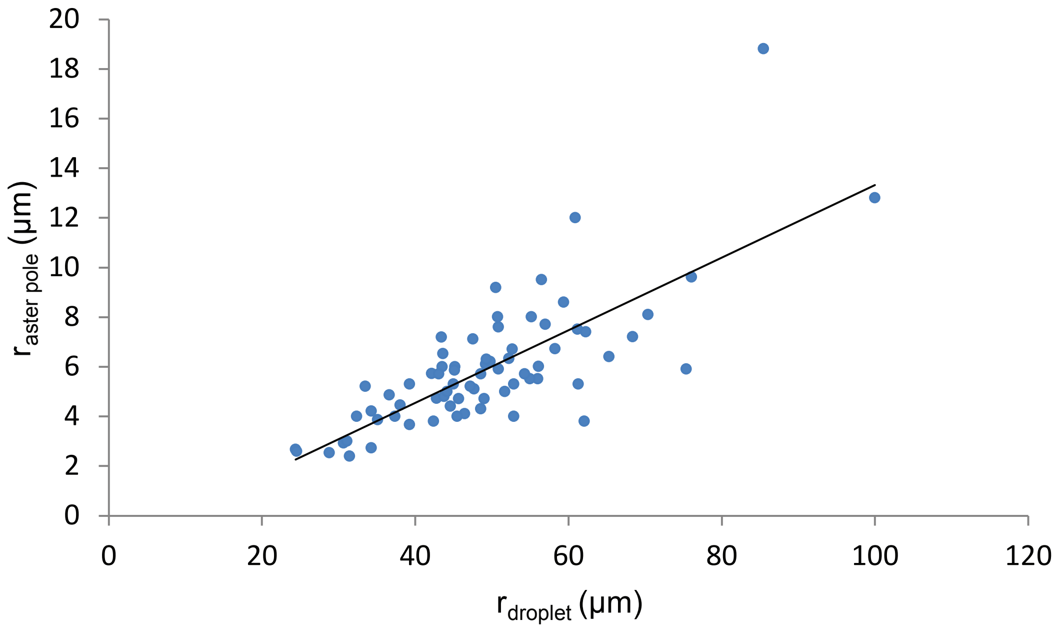

Figure S3. Plot of the radius of the aster pole entrapping the clusters of ferritins as a function of the radius of the droplets of egg extracts.

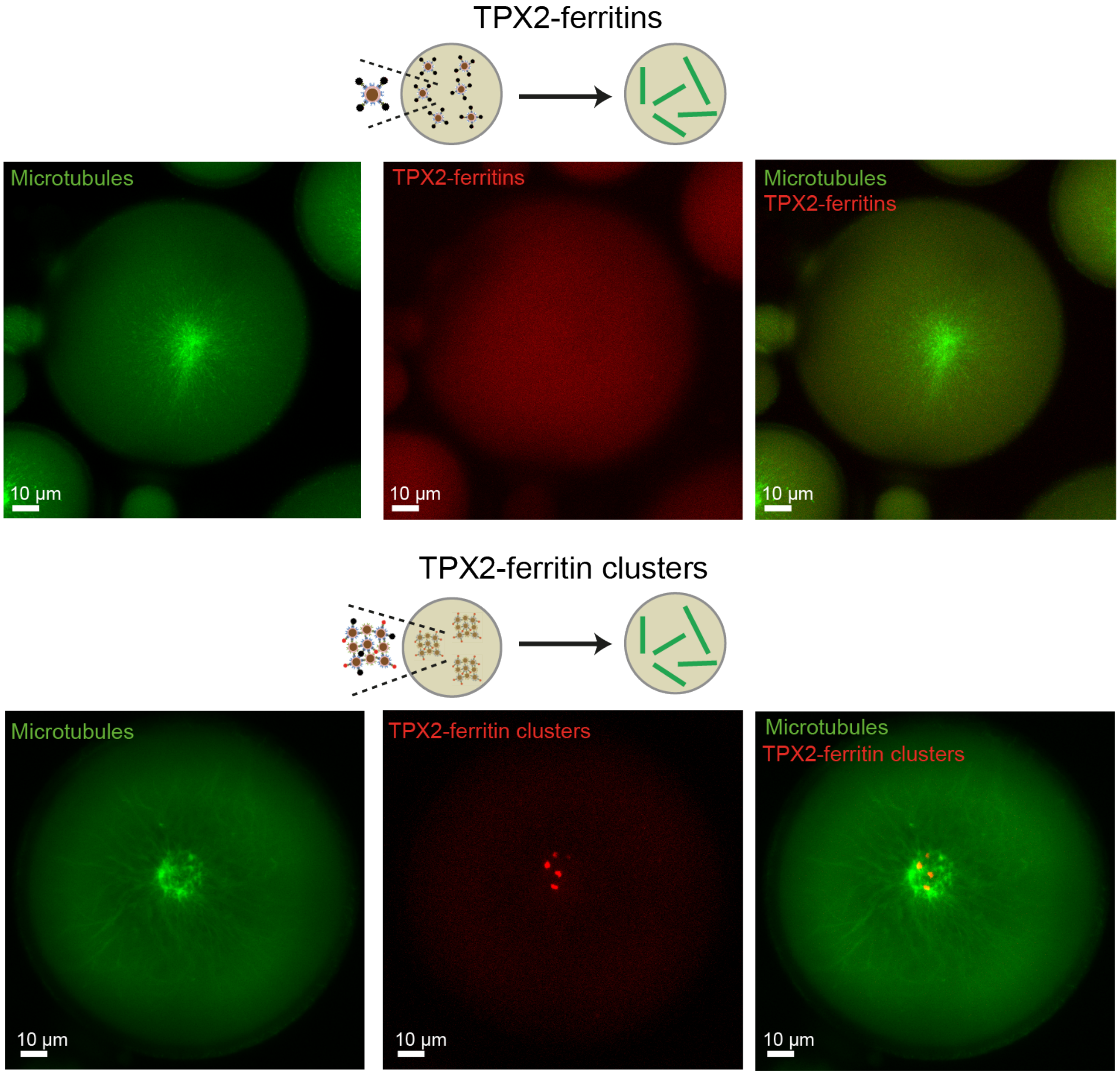

Figure S4. Confocal observation of microtubule polymerization induced by TPX2-ferritins in a droplet of *Xenopus* egg extract (top: freely diffusing TPX2-ferritins, bottom: clusters of TPX2-ferritins).
